# Development of α-Cyclodextrin-Based Orally Disintegrating Tablets for 4-Phenylbutyrate

**DOI:** 10.3390/pharmaceutics16010082

**Published:** 2024-01-07

**Authors:** Kindness L. Commey, Airi Enaka, Ryota Nakamura, Asami Yamamoto, Kenji Tsukigawa, Koji Nishi, Daisuke Iohara, Fumitoshi Hirayama, Masaki Otagiri, Keishi Yamasaki

**Affiliations:** 1 Faculty of Pharmaceutical Sciences, Sojo University, 4-22-1 Ikeda, Kumamoto 860-0082, Japan; g1971d03@m.sojo-u.ac.jp (K.L.C.); g1851033@m.sojo-u.ac.jp (A.E.); g1951079@m.sojo-u.ac.jp (R.N.); g2051122@m.sojo-u.ac.jp (A.Y.); tsukigawa@ph.sojo-u.ac.jp (K.T.); knishi@ph.sojo-u.ac.jp (K.N.); dio@ph.sojo-u.ac.jp (D.I.); fhira@ph.sojo-u.ac.jp (F.H.); 2 DDS Research Institute, Sojo University, 4-22-1 Ikeda, Kumamoto 860-0082, Japan

**Keywords:** cyclodextrins, 4-phenylbutyrate, orally disintegrating tablets, solid-state complexation, pediatric

## Abstract

Despite major improvements brought about by the introduction of taste-masked formulations of 4-phenylbutyrate (PB), poor compliance remains a significant drawback to treatment for some pediatric and dysphagic patients with urea cycle disorders (UCDs). This study reports on the development of a cyclodextrin (CD)-based orally disintegrating tablet (ODT) formulation for PB as an alternative to existing formulations. This is based on previous reports of the PB taste-masking potential of CDs and the suitability of ODTs for improving compliance in pediatric and dysphagic populations. In preliminary studies, the interactions of PB with α and βCD in the solid state were characterized using X-ray diffraction, scanning electron microscopy, dissolution, and accelerated stability studies. Based on these studies, lyophilized PB-CD solid systems were formulated into ODTs after wet granulation. Evaluation of the ODTs showed that they had adequate physical characteristics, including hardness and friability and good storage stability. Notably, the developed αCD-based ODT for PB had a disintegration time of 28 s and achieved a slightly acidic and agreeable pH (≈5.5) in solution, which is suitable for effective PB-CD complexation and taste masking. The developed formulation could be helpful as an alternative to existing PB formulations, especially for pediatric and dysphagic UCD patients.

## 1. Introduction

4-phenylbutyrate (PB) is indicated for the management of urea cycle disorders (UCDs), which are inborn metabolic disorders resulting from deficiencies in the urea cycle and characterized by hyperammonemia [1,2,3]. PB is also under clinical trials for cystic fibrosis, cancer, and hemoglobinopathies [4,5,6].

A significant drawback to the clinical use of PB is its unpleasant taste, which leads to poor compliance, especially in children [7,8]. Although taste-masked PB formulations are available and have helped improve compliance, they do not sufficiently cater to a critical section of the UCD patient population, particularly children under the age of two. This is due to either contraindications in this patient population or inadequacy of the formulations in addressing the dysphagia commonly experienced by patients in this age group. For instance, glycerol PB, though tasteless, requires a competent pancreatic exocrine function, which may be immature in this patient population. Another formulation requires quick swallowing (~10 s) to maintain taste masking, which can be challenging for some patients [9,10,11,12,13].

To address these limitations, we explored cyclodextrin (CD) complexation as an alternative PB taste-masking strategy. CDs are cyclic oligosaccharides comprising six, seven, eight, or more D-glucopyranose units linked by α-1,4-glycosidic bonds that form inclusion complexes with a wide range of molecules. The formation of these complexes offers numerous pharmaceutical benefits, including the improvement in drug solubility and stability and the taste-masking of unpleasant-tasting drugs [14,15,16,17]. Furthermore, CDs typically exhibit low oral bioavailability and are considered practically non-toxic when administered orally [14,18]. From our studies of the interactions between PB and the natural CDs in solution, we recently reported that the CDs, especially αCD, can effectively mask the bitter taste of PB through the formation of inclusion complexes and can address the limitations of current PB market formulations [19].

Our continuing investigations focused on developing CD-based solid oral PB formulations as an alternative to existing market formulations. This aligns with the recent efforts to shift from liquid to solid oral dosage forms for pediatric medicines. This is because liquid formulations face stability issues, particularly in tropical areas, and are expensive to transport and store [20,21,22]. Considering the target section of UCD patients with unmet needs regarding suitable PB formulation for nitrogen control, orodispersible formulations were deemed necessary as conventional tablet or capsule formulations would be inappropriate. A CD-based orally disintegrating tablet (ODT) formulation for PB, which remains stable in the solid state but quickly disintegrates in the mouth to form taste-masked PB solutions or suspensions, would be suitable for these pediatric UCD patients and individuals with dysphagia. Such a formulation would benefit the target population as it would be easier and more convenient to administer and swallow, thereby improving compliance [23,24,25,26,27].

Therefore, the objective of the present study was to develop a CD-based ODT formulation for PB. First, we prepared PB-αCD and PB-βCD solid systems and characterized the interaction between PB and the CDs in the solid state by X-ray diffraction, scanning electron microscopy, and dissolution studies [28,29]. We then formulated and prepared PB-CD ODTs using lyophilized PB-CD solid systems based on our preliminary findings. An αCD-based ODT formulation of PB with adequate physical characteristics, storage stability, and a disintegration time of about 28 s was successfully developed. To our knowledge, this appears to be the first report on the solid-state characterization of interactions between PB and CDs and the development of a CD-based ODT formulation for PB. The developed formulation is designed to address the limitations of current PB formulations and could be helpful as an alternative, especially for pediatric UCD patients and individuals with dysphagia.

## 2. Materials and Methods

### 2.1. Materials

PB (as sodium salt) was purchased from LKT Laboratories Inc. (St. Paul, MN, USA), while αCD, βCD, and D-Mannitol were obtained from Nacalai Tesque Inc. (Kyoto, Japan). Microcrystalline cellulose (PH 101) was obtained from Asahi Kasei Chemicals Corp. (Tokyo, Japan), whereas Crospovidone (Kollidon CL-SF) was obtained from BASF Aktiengesellschaft (Ludwigshafen, Germany). Citric acid and Magnesium stearate were sourced from Wako Pure Ind. Co. (Osaka, Japan). All other materials and chemicals were obtained from commercial sources and were of the highest pharmaceutical or analytical grade.

### 2.2. Preparation of PB-CD Solid Systems

PB-CD solid systems were prepared using PB and αCD or βCD, based on the findings of our previous study on PB-CD complexation in solution, using three distinct methods: kneading, lyophilization, and spray-drying. Physical mixtures were also prepared for comparison.

#### 2.2.1. Physical Mixtures (PM)

PB-CD PMs were prepared by simply mixing equimolar quantities of PB and each CD, previously sieved (75–150 μm), uniformly in an agate mortar.

#### 2.2.2. Kneaded Systems (KN)

Equimolar quantities of PB and each CD were triturated in an agate mortar with a small amount of distilled water. The slurry formed was kneaded for 1 h. An appropriate quantity of distilled water was added during this process to maintain a suitable consistency. The final product was then dried at 25 ± 1 °C for ≥48 h in a digitally controlled desiccator (DCD-PSPS, AS ONE Corp., Osaka, Japan).

#### 2.2.3. Lyophilized Systems (LYO)

Equimolar quantities of PB and each CD were dispersed in 20 mL of distilled water. The resulting samples were shaken for 72 h at 25 ± 0.5 °C and 120 rpm (Multi Shaker MMS-3020 in a temperature control chamber FMC-1000; Eyela Co., Tokyo, Japan). The resulting solutions were frozen and then lyophilized in a freeze dryer (Eyela FDU-1200, Tokyo Rikakikai Co., Tokyo, Japan) for 48 h.

#### 2.2.4. Spray-Dried Systems (SD)

Equimolar quantities of PB and each CD were dispersed in 20 mL of distilled water. The resulting samples were shaken for 72 h at 25 ± 0.5 °C and 120 rpm. The resulting solutions were spray-dried using a laboratory-scale spray dryer (Mini Spray Dryer B-290, Büchi Labortechnik, Flawil, Switzerland) coupled to a Büchi Dehumidifier B-296 in open-loop suction mode under the following conditions: inlet temperature of 100 °C, outlet temperatures of 46–50 °C, aspiration rate of 100% (i.e., 38 m^3^/h), compressed air atomization flow rate set at 40 mm on the rotameter and liquid feed rate of 10% (i.e., 3.5 mL/min). 

The prepared solid systems were sieved (75–150 μm) and stored in a digitally controlled desiccator at 25 ± 1 °C.

### 2.3. Evaluation of PB-CD Solid Systems

#### 2.3.1. Scanning Electron Microscopy *(SEM)*

The particle structure and surface morphology of the PB-CD solid systems were examined using a scanning electron microscope at 5.0 kV (Miniscope TM 3000, Hitachi High-Tech. Corp., Tokyo, Japan). The samples were fixed onto an SEM stub using double-sided carbon sticky tape before imaging at a magnification factor of 200.

#### 2.3.2. X-ray Diffraction (XRD)

X-ray diffraction was carried out on the PB-CD solid systems at room temperature using a Rigaku X-ray diffractometer (RINT Ultima+/PC, Rigaku Corp., Tokyo, Japan) to analyze the crystallinity of the systems. CuKα radiation at 40 mA and 40 kV was applied with an angular increment of 0.02° and covering a 2θ range of 5–30°.

#### 2.3.3. Dissolution Studies

Dissolution studies were conducted using a modified flow-through method in the 1st dissolution fluid (JP1) of the Japanese Pharmacopoeia (prepared by dissolving 2.0 g of sodium chloride in 7.0 mL of hydrochloric acid and water to make 1000 mL with pH 1.2) according to a previous report [30]. A volume of 10 mL of the dissolution medium, maintained at 37 ± 0.5 °C in a glass beaker, was passed through a 25 mm flow-through filter holder (Sartorius Stedim Biotech, Göttingen, Germany) containing an Advantec^®^ polytetrafluoroethylene filter membrane disc (0.1 μm pore size) (Toyo Roshi Kaisha, Tokyo, Japan) and 5 mg of PB or its equivalent amount of the PB-CD solid systems. The 5 mg of PB would achieve a maximum concentration of 0.5 mg/mL in the dissolution medium. This met sink conditions because the solubility of PB in the dissolution fluid at 37 ± 0.5 °C was found to be >1.0 mg/mL in preliminary solubility tests. The dissolution medium was recirculated in a closed-loop configuration at 0.27 mL/min by a peristaltic pump (Perista SJ-1121H, Atto Corp., Tokyo, Japan). As One^®^ pump tubings (3 mm × 5 mm) (AS ONE Corp., Osaka, Japan) were used to connect the various setup parts for each run. At predetermined time intervals, 50 μL samples were withdrawn from the beaker and collected in HPLC vials for assay. To maintain the total liquid volume in the setup, 50 μL of fresh dissolution fluid was immediately added to the beaker for recirculation after each sampling. The measurement was conducted for 30 min. The dissolution studies were performed in triplicate. The dissolution profiles were evaluated by the percent drug dissolved at 2 min (DP_2min_) and the dissolution efficiency at 30 min (DE_30min_), calculated from the area under the dissolution curve according to Equation (1) [31]:

(1) $$Dissolution\;efficiency\;(DE)=\frac{\int_{0}^{t}ydt}{y_{100}\times t}\times 100\%$$

where *y* is the amount of drug dissolved at time *t*.

##### HPLC Conditions

HPLC measurements were carried out according to a previous report using a JASCO HPLC system (Jasco Corp., Tokyo, Japan) [32]. The stationary phase was a YMC-PACK ODS AM 303 column (5 µm, 250 mm × 4.6 mm, YMC Co., Kyoto, Japan) maintained at 40 ± 0.5 °C. The mobile phase comprised of two solvents, A (0.05 M sodium dihydrogen phosphate) and B (0.05 M sodium dihydrogen phosphate and acetonitrile (30:70, *v/v*)), programmed as follows: 0–7 min (30–100% B), 7–10 min (100% B), and 10–15 min (100–30% B) at a flow rate of 1 mL/min. A detection wavelength of 210 nm was used and each sample was monitored for 15 min. The retention time for PB was 8.9 min.

#### 2.3.4. Stability Studies

The storage stability of the PB-CD solid systems was studied using an accelerated stability chamber (Model PTH-400NC-D, NK System, Tokyo, Japan). A sample of each solid system (200 mg) in a glass vial was placed uncovered in the stability chamber operating at 40 ± 1 °C and relative humidity (RH) of 75% ± 2% for 90 days. At the end of the period, the physical appearance of each sample was recorded. The samples were weighed and dried to constant weight in a digitally controlled desiccator at 25 ± 1 °C. The moisture content was calculated as the difference in sample weight before and after drying. The actual PB content of known quantities of the solid systems collected from the top, middle, and bottom of each sample was then determined by HPLC. The HPLC conditions for the assay were identical to those for the dissolution studies. Additionally, degradant analysis was performed by comparing the ^1^H NMR spectra of D_2_O solutions of the solid systems before and after the stability study. X-ray diffraction and dissolution studies were repeated on the dried solid systems according to methods previously described in Section 2.3.2 and Section 2.3.3, respectively.

### 2.4. Formulation of PB-CD ODTs

PB-CD granules were prepared using the wet granulation method with ingredients listed in Table 1. PB or equivalent weights of PB-αCD or PB-βCD lyophilized solid systems were mixed with the excipients, except Magnesium stearate, in a porcelain mortar to obtain a uniform blend. Granulation was performed by adding water dropwise. Granules were obtained after wet sieving (mesh #14) and drying at 55 ± 1 °C for 1 h in a hot air oven (Eyela NDO-500W, Tokyo Rikakikai Co., Japan). The dry granules were sieved again (mesh #45) to obtain a particle size range of 0.355–1.400 mm. The granules were then lubricated by tumbling with pre-sieved Magnesium stearate (mesh #80) in closed glass vials for about 15 min. Accurately weighed quantities of the granules (200 mg) were compressed into tablets using a single-punch rotary tablet press with a 10 mm circular flat punch. (Model P-16B-027B, Riken Seiki Co., Niigata, Japan). A force of 5 kN was used to compress the PB tablets, whereas a force of about 3.5 kN was used for the PB-αCD and PB-βCD tablets. The tablets were stored in a digitally controlled desiccator at 25 ± 1 °C.

### 2.5. Evaluation of PB-CD ODT Formulations

The prepared ODTs were evaluated for weight variation, thickness, hardness and tensile strength, friability, pH in solution, and drug content according to standard procedures. For the weight variation test, the average weight of twenty randomly selected tablets was obtained using a digital weighing balance (Mettler-Toledo, Greifensee, Switzerland). The tablets were then weighed individually, and variations from the average weight were calculated. The thickness of ten randomly selected tablets was measured using a digital caliper (AS ONE Corp., Osaka, Japan), and their hardness was determined using a hardness tester (Kujiwara Seisakusho, Tokyo, Japan). The tensile strength of the tablets was calculated according to Equation (2) [33]:

(2) $$Tensile\;strength\;(MPa)=\frac{2H}{\pi dt}$$

where H is the tablet hardness (N), d is the diameter (mm), and *t* is the thickness (mm). 

Tablet friability was determined using a Toyama friabilator (Toyama Sangyo Co., Osaka, Japan).

#### 2.5.1. pH in Solution

A tablet was powdered, dispersed in 1 mL of 10 mM KCl, and sonicated for 30 min, considering that the average stimulated salivary flow rate is 0.5–1.5 mL/min, with children producing about half the volume of adults [34,35]. The resulting suspensions were filtered through 0.2 µm membrane filters (Minisart RC 4, Sartorius Stedim Lab., Stonehouse, UK), and the pH of the filtrate was measured (Horiba Scientific, Tokyo, Japan). The determination was carried out in triplicate.

#### 2.5.2. Assay

Ten randomly selected tablets were powdered, and a portion corresponding to 10 mg of PB was dispersed in 3 mL of ultrapure water and sonicated for 1 h to extract PB. The resulting suspensions were filtered through 0.2 µm membrane filters and diluted appropriately. The PB content was determined by HPLC, as described in Section 2.3.3. The determination was carried out in triplicate.

#### 2.5.3. Wettability

The wettability of the tablets was determined by measuring the contact angle (wetting angle) of a randomly selected tablet using a contact angle meter (DropMaster DM-501, Kyowa Interface Science Co., Saitama, Japan). The determination was carried out in triplicate.

#### 2.5.4. Water Uptake Behavior

The water uptake behavior of the tablets was evaluated by measuring the time course of water displacement in a polyethylene tubing when placed in contact with one flat side of a tablet as the tablet absorbs the water. The water uptake rate constant and maximum water uptake were estimated, assuming a pseudo-first-order rate. A representation of the experimental setup and the data analysis method are shown in Figure S1. The determination was carried out in triplicate.

#### 2.5.5. Moisture Absorption on Storage

The moisture absorption of the tablets on storage was evaluated using an accelerated stability chamber. Ten randomly selected tablets in glass vials were placed uncovered in the stability chamber operating at 25 ± 1 °C and 75% RH ± 2% RH for 15 days. At the end of the period, the tablets were weighed and dried to constant weight in a digitally controlled desiccator at 25 ± 1 °C. The moisture absorbed was calculated as the difference in tablet weight before and after drying.

#### 2.5.6. Disintegration Time

The disintegration test was carried out using a disintegration tester (HM-21D, Miyamoto Riken Ind. Co., Osaka, Japan). Distilled water at 37 ± 0.5 °C was used as the test medium. A tablet was placed in one of the tubes in the test compartment and agitated at a speed of 31 plunges/min. Disintegration was deemed complete when all particles passed through the screen. The disintegration times of 6 tablets were measured.

### 2.6. Statistical Analysis

Statistical significance was evaluated by the two-tailed paired student’s t-test for comparison between two mean values and by analysis of variance, followed by the Bonferroni test for comparison among more than two mean values. A *p*-value < 0.05 was considered significant.

## 3. Results and Discussion

### 3.1. Evaluation of PB-CD Solid Systems

#### 3.1.1. Morphology and Crystallinity

**Figure 1:**
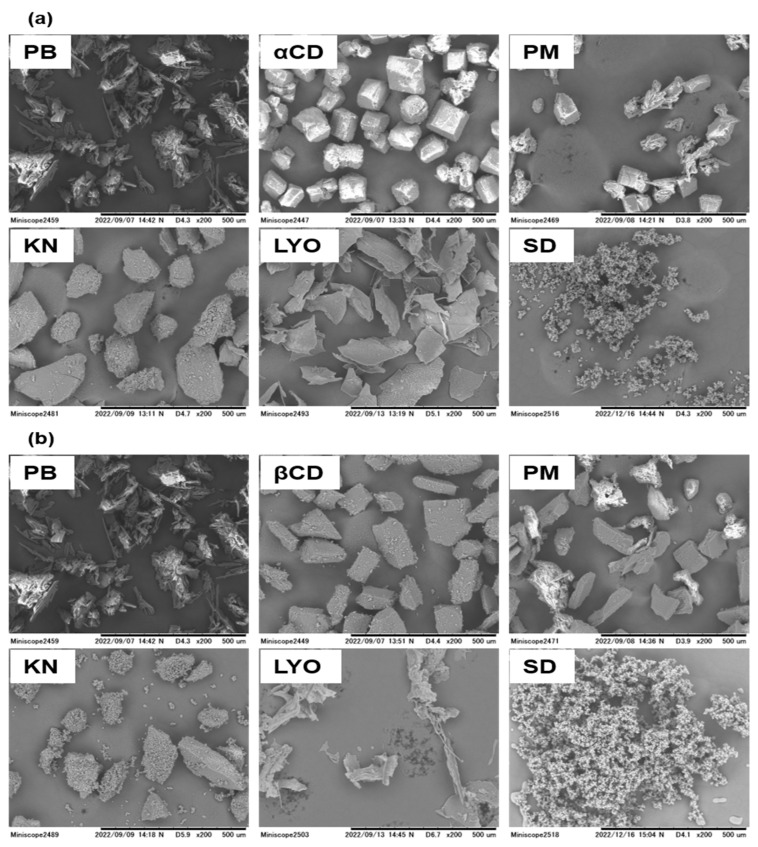
SEM images of PB-αCD (**a**) and PB-βCD (**b**) solid systems in 1:1 PB:CD molar ratio. PM, physical mixture; KN, kneaded; LYO, lyophilized; SD, spray-dried.

The interaction of PB with the CDs in the solid state was examined to provide the basis for designing and formulating CD-based solid formulations for PB. SEM images of the PB-CD solid systems are shown in Figure 1a,b. PB is characterized by clustered crystals, while the CDs present as well-defined polyhedra. The images for the physical mixtures represent a summation of their respective crystalline components, with both the PB and CD crystals visible. In the kneaded systems, polyhedron-shaped particles slightly different from the original shape of the CDs are observed, while the PB crystals are not readily visible. The slight differences in the morphology of the kneaded systems compared to the physical mixtures suggest changes due to the effect of the kneading process or an inefficient complexation by kneading [29]. In contrast, the lyophilized systems present as fluffy sheets, whereas the spray-dried systems appear as homogenous aggregates of spherical particles, with the PB and CD crystals disappearing entirely in both these systems. These suggest the emergence of a single component in the lyophilized and spray-dried systems [28,29].

**Figure 2:**
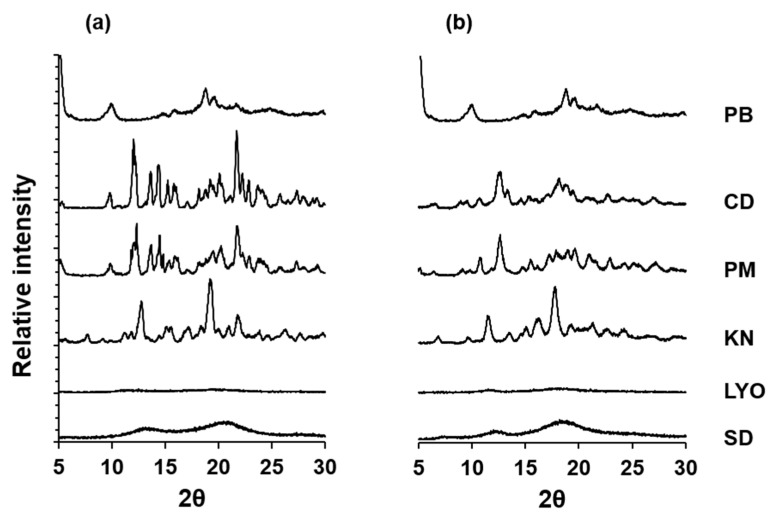
XRD patterns of PB-αCD (**a**) and PB-βCD (**b**) solid systems in 1:1 PB:CD molar ratio. PM, physical mixture; KN, kneaded; LYO, lyophilized; SD, spray-dried.

The XRD patterns of the solid systems, shown in Figure 2a,b, support these observations. PB shows peaks at diffraction angles (2θ) of 9.96, 15.98, 18.78, 19.68, and 21.70°, with the CDs also showing several peaks. The physical mixtures show a combination of the patterns for PB and the respective CDs. The XRD patterns of the kneaded systems are nearly identical to the physical mixtures. However, new peaks at 7.76 and 12.78° for PB-αCD KN (Figure 2a) and 11.48° for PB-βCD KN (Figure 2b) coupled with the increase in the intensity of the PB peak at 18.78° in both kneaded systems are consistent with an inefficient complexation, with free crystalline PB still present [29]. In contrast, no PB or CD peaks are detectable in the lyophilized and spray-dried systems, which show halo patterns, indicating total amorphization. This indicates that PB is not present as a crystalline material in the lyophilized and spray-dried systems, and the amorphous state of these systems may be due to PB-CD solid-state interaction, which suggests the possible formation of inclusion complexes [28,29,36,37].

#### 3.1.2. Dissolution Behavior

**Figure 3:**
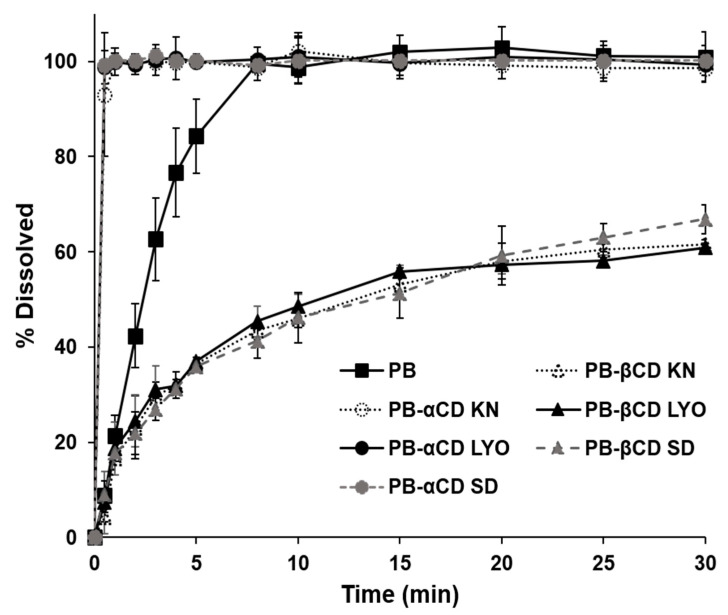
Dissolution profiles of PB-CD (1:1) solid systems in JP 1st dissolution fluid (pH 1.2) at 37 ± 0.5 °C. Each point represents the mean ± SD (*n* = 3). PM, physical mixture; KN, kneaded; LYO, lyophilized; SD, spray-dried.

Dissolution studies were performed to assess the effect of the preparation method and the type of CD on the dissolution behavior of the PB-CD solid systems. The dissolution profiles of the solid systems are shown in Figure 3. In order to evaluate the dissolution profiles, the percent drug dissolved at 2 min (DP_2min_) and the dissolution efficiency at 30 min (DE_30min_) were calculated and are shown in Table 2 [29,31]. The results indicate that the preparation method of the solid systems has no significant impact on their dissolution behaviors. However, αCD significantly increased the dissolution rate (*p* < 0.01), as well as improved the dissolution efficiency (*p* < 0.01) of PB, as evidenced by the DP_2min_ and DE_30min_ values, respectively, of the PB-αCD systems relative to PB. In contrast, βCD significantly slowed the dissolution (*p* < 0.05) and reduced the dissolution efficiency (*p* < 0.001) of PB [29,36]. This could be explained by the solubility of the inclusion complexes formed between PB and the CDs. While PB forms soluble inclusion complexes with αCD, its βCD complexes have limited solubility. Therefore, these results confirm the existence of PB-CD inclusion complexes in the solid systems [19,32].

Since a slow dissolution precludes a PB market formulation for use via nasogastric or gastrostomy tubes, a PB-CD solid system with a faster dissolution rate and improved dissolution efficiency would be desirable to address this limitation [11,13]. Thus, the PB-αCD solid system may be suitable for this application.

#### 3.1.3. Storage Stability

Accelerated stability studies were conducted to evaluate the storage stability of the solid systems as PB is hygroscopic [11]. The physical appearance, moisture, and PB content of the PB-CD solid systems after accelerated stability studies are summarized in Table 3. PB changed color from white to off-white and became caked. However, no noticeable changes in color were observed for the PB-CD solid systems, and they showed less caking compared to PB. The moisture absorbed by PB was reduced from about 30% *w/w* to < 3% *w/w* for the PB-CD solid systems prepared by kneading, lyophilization, and spray-drying. The reduced moisture absorption and better physical appearance of the PB-CD solid systems on storage may be attributable to the protective and stabilizing role of the CDs through a carrier-drug solid-state interaction, indicative of inclusion complex formation. Moreover, the PB content of the systems remained unchanged, and no new peaks were observed in their HPLC chromatograms or ^1^H NMR spectra after the stability studies, indicating no PB decomposition on storage [38,39]. 

**Figure 4:**
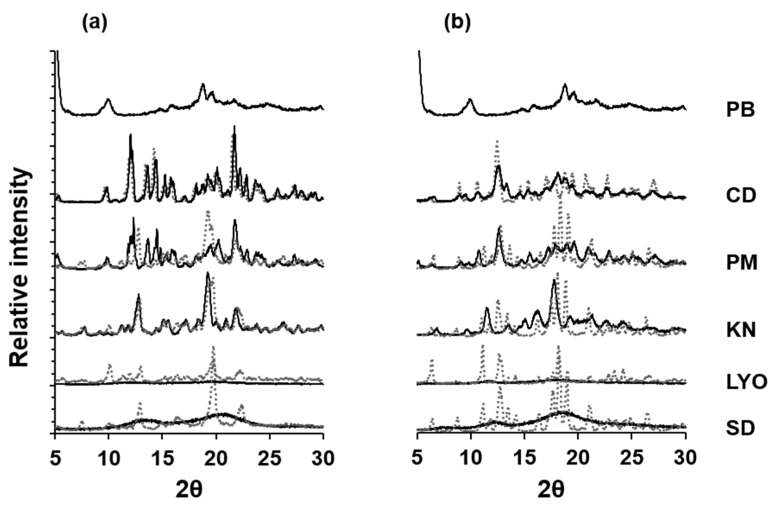
XRD patterns of PB-αCD (**a**) and PB-βCD (**b**) solid systems in 1:1 PB:CD molar ratio before and after storage in an accelerated stability chamber (40 ± 1 °C, 75% RH ± 2% RH, open, for 90 days). Solid line patterns (before); Dotted line patterns (after). PM, physical mixture; KN, kneaded; LYO, lyophilized; SD, spray-dried. The pattern for PB after storage could not be obtained due to the formation of a hardened cake after drying.

A comparison of the XRD patterns of the systems before and after the stability studies is shown in Figure 4a,b. The patterns for PB after the study could not be obtained as a hardened cake was obtained. The PB peaks at 2θ: 18.78, 19.68, and 21.70° appear more intense in all the systems, especially in the PB-βCD systems after stability testing. Since CD-guest inclusion complexes in solution are known to become less stable with increasing temperature, the intensified PB and CD peaks suggest dissociation of the PB-CD complexes. The relatively lower intensities observed in the lyophilized systems, especially the lyophilized PB-αCD system, suggest that less dissociation occurred in those systems, possibly due to reduced moisture absorption [14,38]. Notwithstanding, storage in a tightly sealed container is recommended to enhance stability. There were no significant changes in the dissolution behavior of the solid systems after the stability studies. In addition to the accelerated stability study, a long-term stability study would be necessary to evaluate the stability of the solid systems more thoroughly [38].

### 3.2. Formulation and Evaluation of PB-CD ODTs

PB-CD ODTs were formulated using lyophilized PB-CD solid systems. Although both the lyophilized and spray-dried systems were completely amorphous, suggesting efficient solid-state CD complexation of PB, the lyophilized systems exhibited less dissociation of the complexes to release free bitter PB during storage compared to the spray-dried systems, as shown in Figure 4. Thus, the lyophilized systems were more suitable for preparing taste-masked formulations. The lyophilized PB-αCD and PB-βCD solid systems were prepared based on previously reported drug: CD ratios (i.e., 1:1.18 and 1:1.50 for PB-αCD and PB-βCD, respectively) to enhance complexation efficiency [19,40]. Preliminary attempts were made to formulate the ODTs by direct compression due to the convenience and cost-effectiveness of this method. However, it was unsuccessful in obtaining ODTs of appropriate characteristics since direct compression is highly influenced by the characteristics of the active pharmaceutical ingredient (API) and the excipients used [20,41]. Therefore, the wet granulation method was used to obtain PB-CD ODTs with suitable characteristics.

#### 3.2.1. Physical Characterization of ODTs

A summary of the physical characteristics of the prepared PB-CD ODTs is shown in Table 4. The tablets exhibited low weight variation with good content uniformity, which is crucial to ensuring dosing uniformity. The results indicate a satisfactory manufacturing process and a good quality of the ODTs [20,27]. The CDs appear to impart good compressibility properties to the granules since a lower compression force was required to produce PB-CD ODTs with significantly greater hardness and tensile strength (*p* < 0.001) compared to the tablets with no CD and more mannitol, which is known to have insufficient binding and compressibility properties [20]. The hardness of the PB-CD ODTs is consistent with the values (24.11–39.20 N) reported for ODTs, including CD-based ODTs of identical size [37,39,42]. The prepared PB-CD ODTs thus had sufficient mechanical stability to withstand further processing like packaging and practical handling. Additionally, the ODT formulations showed low friability (<1%), indicating adequate abrasion resistance [37,42]. 

It was necessary to assess the pH of the tablets in solution because PB is ionizable, and a slightly acidic pH (~5.5) is required to ensure effective PB-CD complexation for taste-masking [19]. Citric acid (1.5% *w/w*) was included in the ODT formulations to achieve this desired pH. The PB-αCD ODT achieved an ideal pH of 5.42, while the pH of the PB-βCD ODTs (6.08) was slightly higher than desired.

#### 3.2.2. Water Uptake and Disintegration Performance of ODTs

**Figure 5:**
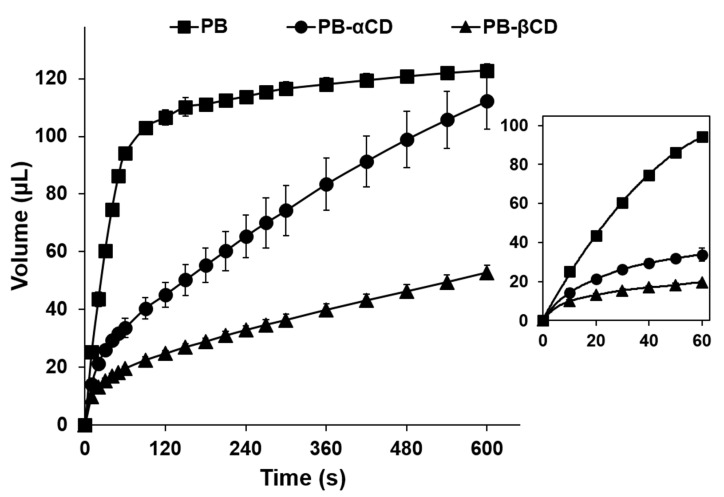
Water uptake behavior of PB-CD ODTs. Each point represents the mean ± SD (*n* = 3). The inset shows the water uptake of the formulations during the initial 60 s.

The water uptake and disintegration performance of the prepared ODTs were evaluated to assess the effect of the CDs on these parameters, as shown in Table 5. Tablet wettability refers to how well a tablet can be wetted by a liquid, such as water or saliva. The contact angle (wetting angle) of the tablets suggests that the CDs (which replaced much of the more porous mannitol) decreased the wettability of the tablets. In particular, the PB-βCD ODTs showed a significantly decreased wettability (*p* < 0.05) compared to the PB ODTs, indicating reduced porosity [43]. Moreover, the water uptake behavior of the tablets, as shown in Figure 5, indicates a faster water uptake and a significantly higher maximum water uptake (V_max_) (*p* < 0.001) for the PB-αCD ODTs compared to the PB-βCD ODTs. 

A tablet’s wettability and water uptake can affect its storage stability. The moisture absorption of the tablets during storage was therefore evaluated, as ODTs are often hygroscopic under normal conditions of temperature and humidity, especially if the API, like PB, is also hygroscopic [11,42]. The results show that the PB-CD ODT formulations had significantly lower moisture absorption during storage (*p* < 0.001) compared to the PB ODTs, with both PB-CD ODTs having a water absorption of <2% *w/w*, indicating good storage stability [27]. 

Tablet wettability and initial water uptake rate also affect disintegration [43,44]. In addition to the pores resulting from the granulation process, the disintegration of the prepared ODTs would depend on water uptake by the included crospovidone and microcrystalline cellulose, which swell and burst when they come in contact with saliva, creating channels in the tablet matrix [44]. The results, therefore, suggest that the PB-αCD ODT would disintegrate faster than the PB-βCD ODT. The US Food and Drug Administration (FDA) recommends a disintegration time of 30 s or less for ODTs based on the USP disintegration test or other equivalent disintegration test methods [45]. The disintegration tests on the formulations indicated that the CDs slowed their disintegration. The disintegration time for the PB-αCD ODT (28.17 s) was within the FDA’s recommendation. On the other hand, the PB-βCD ODT formulation took longer (93.67 s), despite containing the same amount of superdisintegrants and being prepared under identical conditions. It is worth noting, however, that the mannitol content of the ODTs correlates negatively with their disintegration times, possibly due to the improved wettability and water uptake imparted by the soluble and porous mannitol [43,44]. Thus, the superior complexation efficiency of αCD for PB, which allows for more mannitol to be incorporated in the PB-αCD ODTs, gives it an advantage over βCD in this respect [19,32]. However, the PB-βCD ODT formulation meets the requirement of the European Pharmacopoeia, which specifies a limit of 3 min for ODTs [23,27].

The developed αCD-based ODT formulation is designed to have good patient acceptability through the contributions of its component ingredients and excipients [27,46]. These include the PB taste-masking ability of αCD through complexation at the slightly acidic and agreeable pH [19]. Additionally, the palatability of the formulation would be enhanced by the sweetness of mannitol, the smooth texture and excellent mouthfeel imparted by Kollidon CL-SF and microcrystalline cellulose PH 101, and the unique flavor of citric acid [20,42,47,48]. Crucially, the developed formulation satisfies critical criteria for pediatric medicines, with an emphasis on ease of administration, palatability, and the use of safe, well-established, and stable excipients [49].

On the other hand, the βCD-based ODT formulation would require further optimization to improve its characteristics, including disintegration time and pH in solution, as it can also provide an alternative to existing PB formulations. Therefore, the future direction of this study would involve optimizing the developed formulations, including conducting long-term stability studies on the solid systems and the ODTs and employing the more cost-effective direct compression method for preparing the ODTs. Furthermore, human taste evaluation and bioequivalence studies on the optimized formulations will be conducted. Nevertheless, the βCD-based PB granules prepared in this study can be administered directly, or the lyophilized PB-βCD solid system as an extemporaneously prepared suspension in a suitable oral suspension vehicle like Ora-Plus, which is buffered to a pH of about 4.2 [50]. Taste-masking in these cases is expected to be enhanced by the decreased PB solubility through complexation with βCD [51].

## 4. Conclusions

The present study explored the preparation of a solid oral CD-based PB formulation based on previous reports indicating that CDs form inclusion complexes with PB in solution and can be used to mask its unpleasant taste. Since children constitute a significant and critical population of UCD patients, the study focused on preparing an ODT formulation. PB-CD solid systems with good dissolution profiles and storage stability were prepared by lyophilization. An αCD-based ODT formulation of PB with adequate physical characteristics, storage stability, and a disintegration time of 28 s was formulated and prepared using the PB-αCD solid system. The developed αCD-based ODT achieved a slightly acidic pH of about 5.5 in solution, which is suitable for effective PB-CD complexation and taste masking. The developed formulation could benefit pediatric UCD patients and individuals with dysphagia as an alternative to existing formulations.

## Figures and Tables

**Table 1:** Formulae for PB-CD ODTs.

Ingredients	Weight (mg)		
	PB	PB-αCD	PB-βCD
PB (or equivalent PB-CD solid system)	10.00	71.70 ^a^	101.30 ^a^
Microcrystalline cellulose (PH 101)	10.00	10.00	10.00
Crospovidone (Kollidon CL-SF)	10.00	10.00	10.00
D-Mannitol	165.00	103.30	73.70
Citric acid	3.00	3.00	3.00
Magnesium stearate	2.00	2.00	2.00
Total	200.00	200.00	200.00

^a^ According to the Drug: CD ratios [19].

**Table 2:** Dissolution parameters of PB-CD solid systems in JP 1st dissolution fluid (pH 1.2) at 37 ± 0.5 °C.

	PB	PB-αCD			PB-βCD		
		KN	LYO	SD	KN	LYO	SD
DP_2min_ (%)	42.40 ± 6.71	100.00 ± 0.71 **	99.40 ± 2.09 **	100.20 ± 1.27 **	23.15 ± 6.62 *	24.47 ± 5.46 *	21.87 ± 4.50 *
DE_30min_ (%)	91.83 ± 1.83	99.27 ± 2.94 **	99.41 ± 1.02 **	99.34 ± 0.06 **	48.49 ± 1.74 ***	49.05 ± 0.17 ***	48.86 ± 3.08 ***

DP_2min_; percent PB dissolved at 2 min, DE_30min_; dissolution efficiency (area under the dissolution curve) at 30 min. * *p* < 0.05, ** *p* < 0.01, *** *p* < 0.001 vs. PB solid system (*n* = 3). PM, physical mixture; KN, kneaded; LYO, lyophilized; SD, spray-dried.

**Table 3:** Physical appearance, moisture content, and PB content of PB-CD solid systems after open storage in an accelerated stability chamber at 40 ± 1 °C and 75% RH ± 2% RH for 90 days.

Solid System	Physical Appearance	Moisture Content (% *w/w*)	PB Content (% *w/w*) ^a^
PB	Color change from white to off-white. Complete caking with no free flow.	30.45	100.33 ± 3.41
αCD	No color changes. Slight to moderate caking with limited flow.	2.73	-
PM		4.14	98.19 ± 4.25
KN		2.14	101.47 ± 1.39
LYO		1.36	99.65 ± 2.56
SD		2.36	98.95 ± 1.56
βCD	No color changes. Slight to moderate caking with limited flow.	4.50	-
PM		2.32	98.62 ± 2.73
KN		1.50	99.12 ± 0.73
LYO		0.91	98.77 ± 0.60
SD		1.82	100.04 ± 1.07

^a^ PB content expressed as % of the expected (theoretical) value. The values are mean ± SD (*n* = 3) of samples taken from different depths of each solid system. PM, physical mixture; KN, kneaded; LYO, lyophilized; SD, spray-dried.

**Table 4:** Physical characterization of PB-CD ODTs.

Formulation	Weight (mg) ^a^	Thickness (mm) ^b^	Hardness (N) ^b^	Tensile Strength (MPa) ^b^	Friability (% *w/w*)	pH in solution ^c,d^	Assay (% *w/w*) ^c^
PB	199.78 ± 0.12	1.94 ± 0.03	4.40 ± 0.52	0.14 ± 0.01	0.59	4.88 ± 0.02	96.85 ± 3.09
PB-αCD	200.00 ± 0.18	2.05 ± 0.10	33.30 ± 1.16 ***	1.03 ± 0.02 ***	0.79	5.42 ± 0.03	100.33 ± 1.89
PB-βCD	200.10 ± 0.16	2.06 ± 0.08	26.00 ± 1.41 ***	0.80 ± 0.02 ***	0.85	6.08 ± 0.13	102.52 ± 0.92

^a^: *n* = 20, ^b^: *n* = 10, ^c^: *n* = 3, ^d^ in 1 mL of 10 mM KCl, *** *p* < 0.001 vs. PB ODT.

**Table 5:** Water uptake and disintegration performance-related parameters of PB-CD ODTs.

Formulation	Wettability (Contact Angle, θ) (°) ^a^	Maximum Water Uptake, V_max_ (µL) ^a^	Moisture Absorption on Storage (% *w/w*) ^b,d^	Disintegration Time (s) ^c^
PB	10.40 ± 1.55	120.50 ± 1.35	2.64 ± 0.08	13.83 ± 2.52
PB-αCD	23.97 ± 5.35	160.63 ± 9.81 ^###^	1.83 ± 0.12 ***	28.17 ± 1.73
PB-βCD	39.07 ± 5.58 *	77.21 ± 3.87 **	1.52 ± 0.03 ***	93.67 ± 5.96

^a^: *n* = 3, ^b^: *n* = 10, ^c^: *n* = 6, ^d^ storage condition was 25 ± 1 °C, 75% RH ± 2% RH, open, for 15 days, * *p* < 0.05, ** *p* < 0.01, *** *p* < 0.001 vs. PB ODT, ^###^ *p* < 0.001 vs. PB-βCD ODT.

## Data Availability

The datasets used or analyzed in this study are available from the corresponding authors on reasonable request.

## Supplementary Materials

The following supporting information can be downloaded at: https://www.mdpi.com/article/10.3390/pharmaceutics16010082/s1, Figure S1: Experimental setup and data analysis method for the water uptake behavior of PB-CD ODTs.
