# Supplementary material for: Development of α-Cyclodextrin-Based Orally Disintegrating Tablets for 4-Phenylbutyrate
Source: Pharmaceutics. 2024 Jan 7;16(1):82. doi: 10.3390/pharmaceutics16010082 (PMC10818935; doi:10.3390/pharmaceutics16010082)
Supplement: Supplementary file 1 [file pharmaceutics-16-00082-s001.zip › pharmaceutics-2808663-supplementary.pdf]

Development of α-Cyclodextrin-Based Orally Disintegrating Tablets for 4-Phenylbutyrate

Kindness L. Commey [1,2], Airi Enaka [1], Ryota Nakamura [1], Asami Yamamoto [1], Kenji Tsukigawa [1,2], Koji Nishi [1,2], Daisuke Iohara [1,2], Fumitoshi Hirayama [1,2], Masaki Otagiri [1,2,*] and Keishi Yamasaki [1,2,*]

[1] Faculty of Pharmaceutical Sciences, Sojo University, 4-22-1 Ikeda, Kumamoto 860-0082, Japan; g1971d03@m.sojo-u.ac.jp (K.L.C.); g1851033@m.sojo-u.ac.jp (A.E.); g1951079@m.sojo-u.ac.jp (R.N.); g2051122@m.sojo-u.ac.jp (A.Y.); tsukigawa@ph.sojo-u.ac.jp (K.T.); knishi@ph.sojo-u.ac.jp (K.N.); dio@ph.sojo-u.ac.jp (D.I.); fhira@ph.sojo-u.ac.jp (F.H.)

[2] DDS Research Institute, Sojo University, 4-22-1 Ikeda, Kumamoto 860-0082, Japan

* Correspondence: otagirim@ph.sojo-u.ac.jp (M.O.); kcyama@ph.sojo-u.ac.jp (K.Y.).

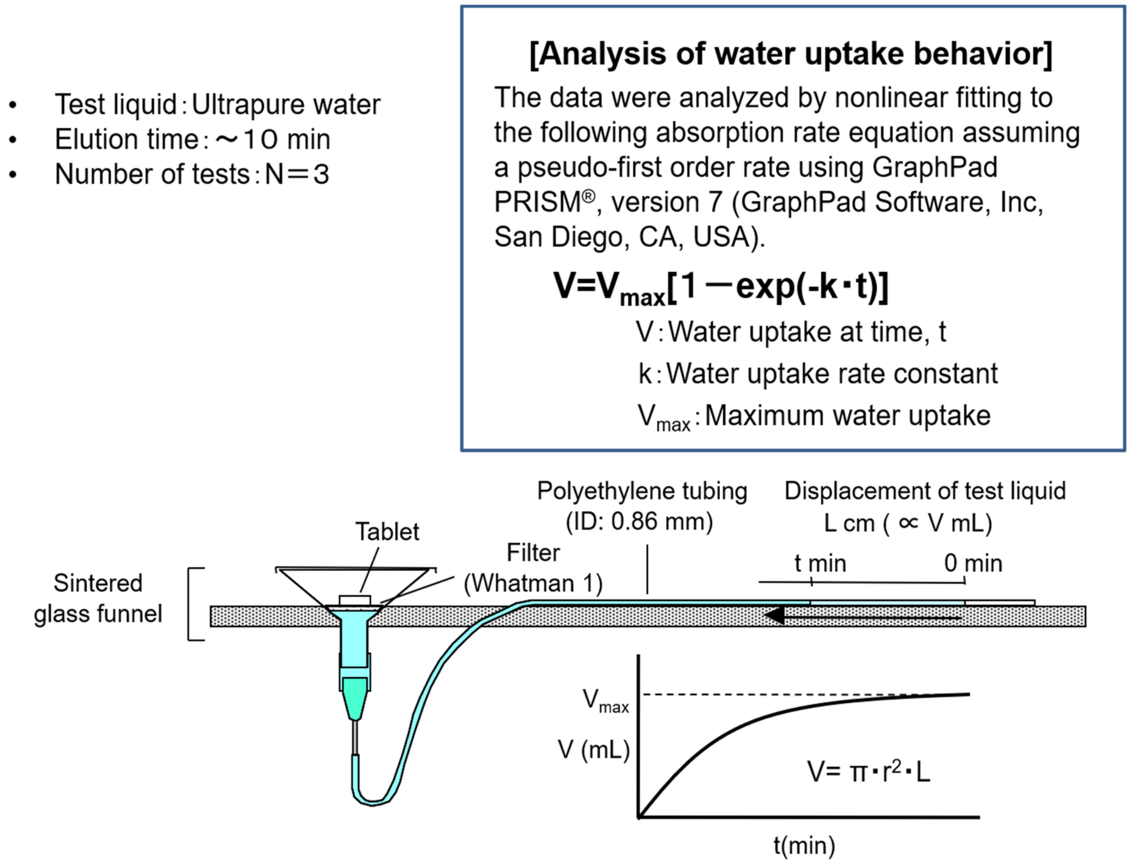

Figure S1. Experimental setup and data analysis method for the water uptake behavior of PB-CD ODTs.
